# *MIR210HG* Accelerates the Progression of Colorectal Cancer and Affects the Function of Colorectal Cancer Cells by Downregulating miR-1226-3p

**DOI:** 10.5152/tjg.2024.23669

**Published:** 2024-12-01

**Authors:** Chunyan Jiang, Xiaofeng Zhao

**Affiliations:** Department of Gastrointestinal Tumor Surgery, Xingtai People’s Hospital, Xingtai, China

**Keywords:** Colorectal cancer, MIR210HG, miR-1226-3p, diagnosis, prognosis

## Abstract

**Background/Aims:**

Colorectal cancer (CRC) is a widespread cancerous disease with an unfavorable prognosis. *MIR210HG* appears to have a significant connection with the development of CRC, but the precise regulatory mechanism remains obscure.

**Materials and Methods:**

Quantitative real-time polymerase chain reaction was utilized to determine expression quantities of *MIR210HG* and miR-1226-3p. The proliferative capacity of CRC cells was measured by cell counting kit-8. The apoptosis rate of cells was examined using flow cytometry. The invasive capability was assessed through the transwell experiment. The targeted regulation of *MIR210HG* and miR-1226-3p was validated through dual-luciferase reporter gene experiments.

**Results:**

In carcinoma tissues and blood serum of colorectal cancer patients and cell lines, *MIR210HG* expression was upregulated, while the miR-1226-3p expression was downregulated. *MIR210HG* had a diagnostic and prognostic value for CRC patients. *MIR210HG* may target and regulate miR-1226-3p. *MIR210HG* may have the capacity to augment the vitality and invasion of CRC cells and suppress cell apoptosis, and this influence is counteracted by miR-1226-3p.

**Conclusion: l:**

ncRNA *MIR210HG* accelerated the progression of colorectal cancer by controlling miR-1226-3p. lncRNA MIR210HG/miR-1226-3p may potentially serve as therapeutic targets for addressing colorectal cancer.

**Main Points**

- *MIR210HG* has a diagnostic value for colorectal cancer (CRC) patients and significantly correlates with the clinical pathological characteristics of CRC.
- *MIR210HG* has the capacity to augment the vitality and invasion of CRC cells and suppress cell apoptosis.
- Inhibition of miR-1226-3p counteracted the influence of *MIR210HG* silencing on CRC cells.

## Introduction

Colorectal cancer (CRC) ranks as the fourth highest contributor to cancer-related deaths globally, leading to approximately 900 000 annual fatalities.^1^ The likelihood of CRC is amplified by undesirable risk factors, including obesity, inadequate physical activity, and tobacco usage, aside from the aging population and dietary patterns in high-income regions.^1^ Reportedly, approximately 50% of CRC patients experience metastasis at the time of diagnosis or after treatment, leading to a poorer prognosis.^2^ Identifying the molecular pathways underlying the progression and metastasis of CRC can help establish novel therapeutic strategies for this disease.

Long non-coding RNAs (lncRNAs) are involved in the control of numerous biological phenomena, including cell growth and differentiation, cell cycle progression, and apoptosis. Disturbance in lncRNA expression has been noted to correlate with the progression of CRC.^3,4^ The lncRNA *MIR210HG* is a gene host that encodes miR-210, which is located at 21q13.3 and consists of 567 nucleotides.^5^ Earlier investigations have revealed that *MIR210HG* serves as an oncogene in a variety of cancerous diseases, promoting tumor progression.^6,7^ However, the functional mechanism in colorectal cancer has been rarely reported.

MicroRNAs (miRNAs) are essential regulators of gene expression.^8^ miRNA also contributes significantly to the development of tumors.^9-11^ Moreover, it has been demonstrated to participate in the development of colorectal cancer.^12,13^ miR-1226-3p serves a vital function in numerous diseases; for instance, it augments the susceptibility of hepatocellular carcinoma to sorafenib by negatively regulating DUSP4 expression.^14^ Previous studies have demonstrated that the lncRNA *MIR210HG* enhances tumor dissemination by functioning as a miR-1226-3p sponge to modulate mucin-1c in invasive breast cancer.^15^ Nonetheless, the mechanism of *MIR210HG* regulating miR-1226-3p in colorectal cancer remains obscure.

This study was the first to investigate the mechanism of *MIR210HG*’s involvement in colorectal cancer and its effect on the function of colorectal cancer cells. Moreover, the targeting effect of *MIR210HG* and miR-1226-3p in colorectal cancer was elucidated for the first time.

## Materials and methods

### Patients and Tissues

A total of 120 colorectal cancer patients from Xingtai People’s Hospital Hospital were recruited, including 45 female patients and 75 male patients, with ages spanning from 25 to 75, and the clinical information of each patient was obtained. No antitumor therapy had been administered to any of the patients before surgery, including radiotherapy and chemotherapy. Seventy-eight healthy patients aged between 25 and 75 years were selected as the control group. The patient’s tumor tissue was collected during the procedure and, at the same time as the tumor tissue was removed, a piece of the patient’s normal colorectal tissue near the patient’s tumor was also collected. This tissue was immediately placed in liquid nitrogen, and the collected samples were rapidly stored in liquid nitrogen and maintained at −80°C. All participants submitted a signed consent form. The study was granted ethical approval by the committee of Xingtai People’s Hospital Hospital (approval no: 2016-009, date: 09 March 2016). In addition, for investigations involving human subjects, informed consent has been obtained from the participants involved.

### Cell Lines and Cell Culture

The human CRC cell lines were sourced from the Shanghai Institute of Biochemistry and Cell Biology. HIEC-6 (normal human intestinal epithelial) cells were acquired from the American Type Cell Culture (ATCC, Manassas, USA). The cells were cultured in RPMI-1640 medium (Gibco, Waltham, USA) supplemented with 10% fetal bovine serum (FBS; Gibco, Waltham, USA) at a temperature of 37°C in a 5% CO_2 _atmosphere.

### Cell Transfection

Logarithmic phase growth-stage cells were plated into 24-well plates. According to the instructions, small interfering RNA (siRNA) directed against *MIR210HG* (si-MIR210HG), or its corresponding control (si-NC) were provided by Sangon Biotech (China). The following is the sequence of si-MIR210HG: 5’-GGGAUUUGGUUCACCUGAATT-3’. si-MIR210HG, si-NC, inhibitor of miR-1226-3p, or its corresponding control (inhibitor-NC) were introduced into CRC cells via Lipofectamine 2000 transfection agent and incubated at a temperature of 37°C in a 5% CO_2_ environment for a duration of 24 hours.

### Quantitative Real-time Polymerase Chain Reaction

Total RNA extraction was performed using Trizol agent (from Invitrogen, Carlsbad, CA, USA), followed by cDNA synthesis utilizing a Reverse Transcription Kit (HaiGene, Harbin, China). RT-PCR was carried out with a Takara SYBR Green PCR reagent kit (Takara, Japan). The primer sequences: GAPDH: Forward: 5’-GCACCGTCAAGGCTGAGAAC-3’. Reverse: 5’-TGGTGAAGACGCCAGTGGA-3’. MIR210HG: Forward: 5’-GGTTCTGGCTTGCTGACAC-3’. Reverse: 5’-CAACTCGGCTTGGTTATTTC-3’. miR-1226-3p: Forward: 5’-GCCGAGTCACCAGCCCTGTG-3’. Reverse: 5’- CTCAACTGGTGTCGTGGA -3’.

### Cell Counting Kit-8 Assay

In order to assess the viability of cells, an experiment utilizing the cell counting kit-8 (CCK-8) was conducted. The cells in the logarithmic growth stage were inoculated onto the 96-well plate and allowed to adhere, according to the instructions. At predetermined time points of 0 hours, 24 hours, 48 hours, and 72 hours, 10 μL of CCK-8 reagent was added to each well. To prevent photodestruction, the reagent was added in a dark environment, and the plates were then returned to the culture incubator for an additional 2-4 hour incubation period. Afterward, the absorbance at 450 nm was determined using an enzyme-labeled device. The experiment was replicated 3 times to ensure statistical significance, and the mean absorbance value was calculated as the final result.

### Detection of Cell Apoptosis Using a Flow Cytometer

The cells of each group in the logarithmic growth phase underwent treatment with trypsin without ethylene diamine tetraacetic acid (EDTA), and the supernatant was discarded after centrifugation. 0.5 mL of staining buffer was added to resuspend the cells, then 5 μL of fluorescein isothiocynate (FITC) dye solution and 5 μL of propidium iodide (PI) dye solution were incorporated into the centrifuge tube. The mixture was thoroughly mixed, and the cell apoptosis was examined using a flow cytometer after standing at ambient temperature for 15 minutes.

### Transwell Assay

Under a 37°C condition, the transwell chamber underwent pre-treatment with 30 μg matrigel for 30 minutes to form a reconstructed basement membrane. The cells were resuspended using RPMI-1640 medium without fetal bovine serum and inoculated into the upper chamber of the transwell. A volume of 600 μL containing RPMI-1640 medium supplemented with 10% fetal bovine serum was introduced into the lower chamber. Following incubation for a period of 48 hours, the cells were preserved in 4% formalin for a duration of 30 minutes, subsequent to which they were treated with crystal violet for a duration of 15 minutes. The non-invasive cells were carefully wiped from the transwell surface with a damp cotton swab, followed by counting the invasive cells under a microscope.

### Dual-luciferase Reporter Assay

The MIR210HG-WT and MIR210HG-MUT reporter vectors were constructed using the pmirGLO reporter vector. Lipofectamine 2000 was employed to transfer the vectors into cells treated with a miR-1226-3p mimic or miR-1226-3p inhibitor. After a transfection duration of 48 hours, the luciferase value was ascertained with the Dual-Luciferase Assay Kit (Yubo).

### Statistical Analyses

The data were displayed as mean ± SD and examined using an independent sample *t*-test, one-way analysis of variance (ANOVA), or 2-way ANOVA. Statistical assessments were performed using GraphPad Prism version 9.0 software (GraphPad Software, Inc., La Jolla, CA, USA). The *χ* ^2^ test was employed to assess the correlation between *MIR210HG* expression and the pathological parameters of CRC patients. Receiver operating characteristic (ROC) curves were employed to assess the diagnostic potential of *MIR210HG* in CRC. A Kaplan–Meier analysis was conducted to investigate the correlation between *MIR210HG* expression and the overall survival of CRC patients. Cox regression analysis was utilized to determine the contributing factors affecting the prognostic status of colorectal cancer patients. A *P*-value below .05 was deemed statistically significant.

## Result

### The Expression Levels and Correlation of *MIR210HG* and miR-1226-3p

**Figure 1.**
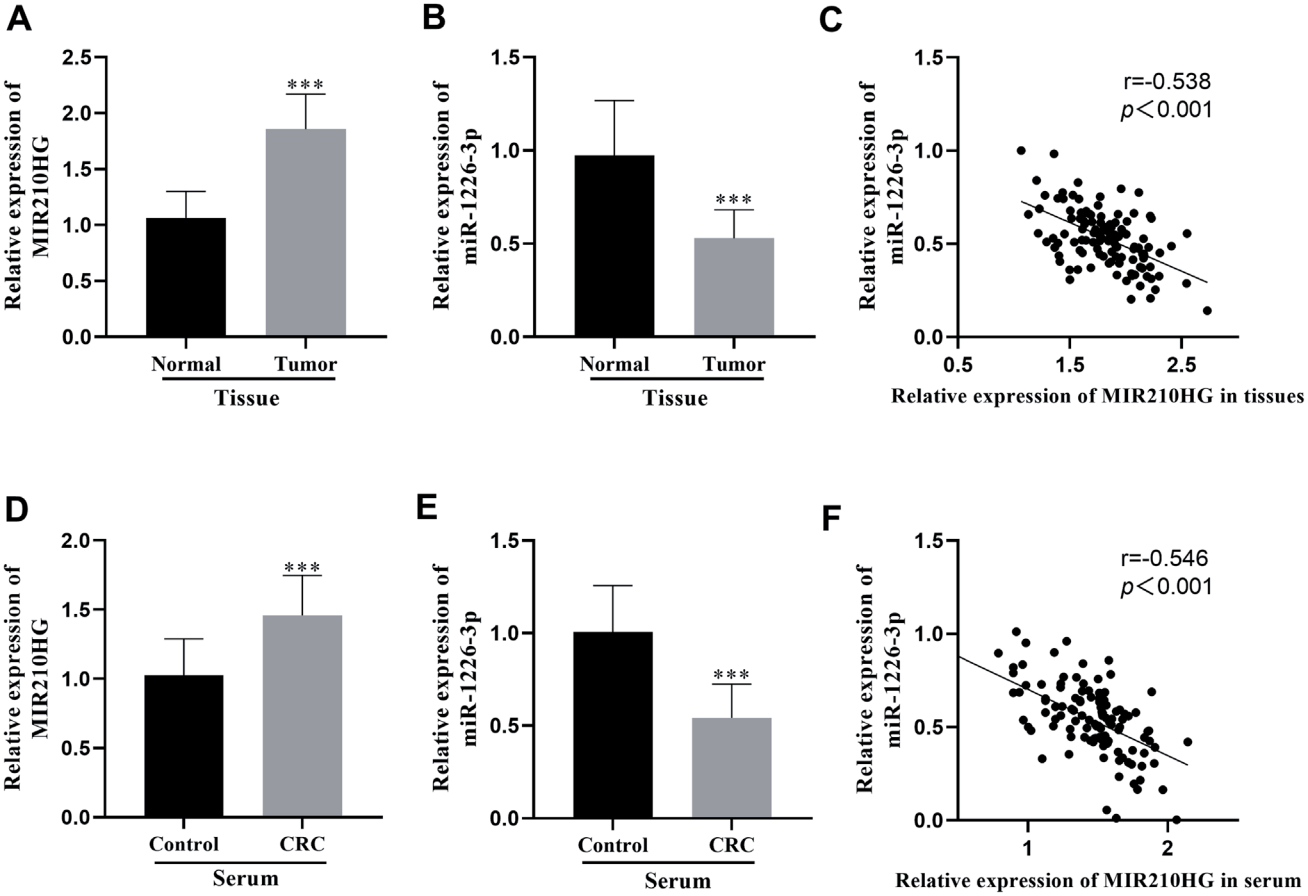
Representation of *MIR210HG* and miR-1226-3p expression and correlation in CRC tissues and cells. (A) The *MIR210HG* expression in CRC tumor tissues was upregulated. (B) miR-1226-3p expression in tumor tissues was downregulated. (C) MIR210HG and miR-1226-3p in CRC tumor tissues were negatively correlated. (D) *MIR210HG* expression was elevated in the serum of CRC. (E) miR-1226-3p expression was suppressed in the serum of CRC. (F) *MIR210HG* and miR-1226-3p were negatively correlated in the serum of CRC.^ ***^P < .001.

The findings indicated that versus the normal tissues of CRC patients, the *MIR210HG* expression level in tumorous tissues was notably upregulated, as illustrated in Figure 1A. The miR-1226-3p expression was reduced in the tumor tissues (Figure 1B). In comparison to healthy subjects, *MIR210HG* in the serum of CRC patients demonstrated a significant elevation (Figure 1D), and the miR-1226-3p level in the serum was significantly decreased (Figure 1E). Whether in tumor tissues or serum, *MIR210HG* and miR-1226-3p showed a negative correlation (Figure 1C and  F).

### *MIR210HG* is Significantly Correlated with the Clinical Pathological Characteristics of Colorectal Cancer

By examining the relationship between *MIR210HG* expression and the clinical pathological aspects of CRC through the Chi-square test, CRC patients were categorized into high and low expression groups according to their average expression levels of *MIR210HG* in tissues and serum. The investigation revealed that when *MIR210HG* exhibited elevated expression in cancerous tissues, more patients presented with lymph node metastasis, tumor stage III-IV, tumor tissue diameter of 4 cm or greater, poor or moderate tumor differentiation, and a high invasion degree (Table 1). When serum *MIR210HG* expression was high, a larger number of patients had a serum carcinoembryonic antigen (CEA) concentration of 5 ng/mL or higher. Moreover, a higher proportion of patients had lymph node metastasis, tumor stage III-IV, tumor tissue diameter of 4 cm or greater, poor or moderate tumor differentiation, and a high invasion degree (Table 2).

### The Diagnosis and Prognosis of Serum *MIR210HG* Expression in Colorectal Cancer

**Figure 2:**
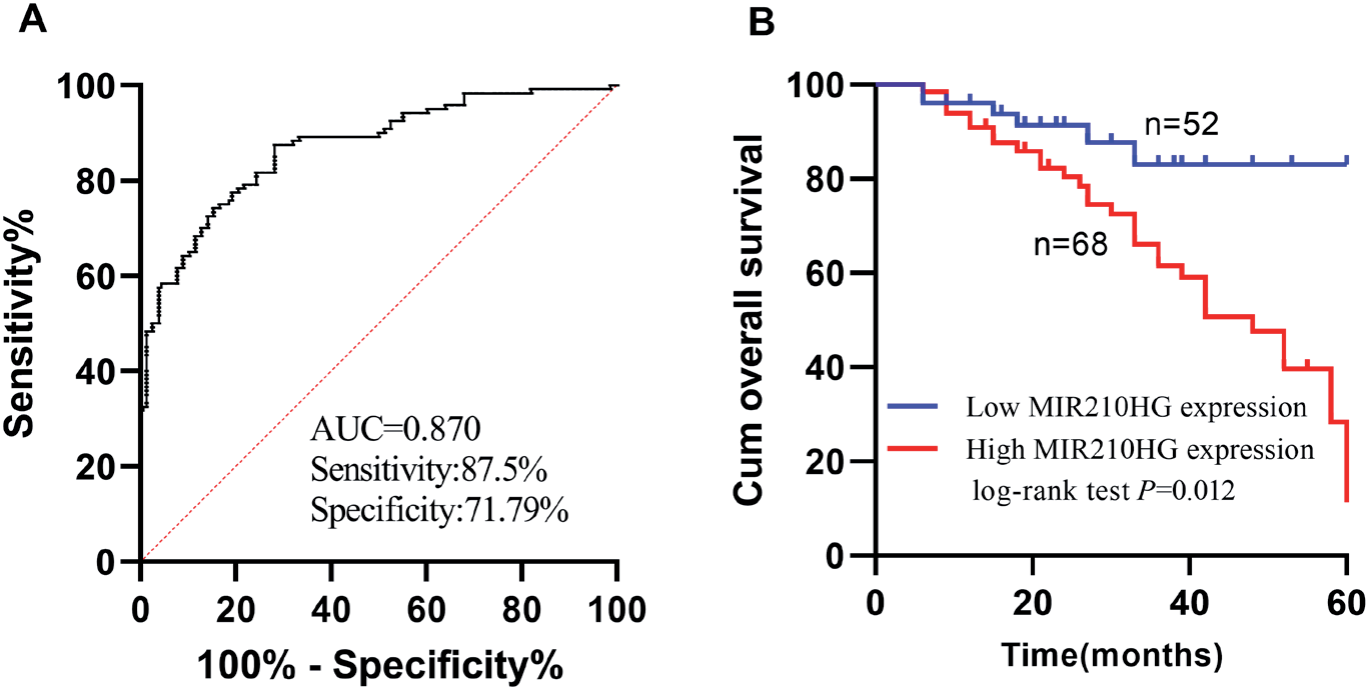
Receiver operating characteristic (ROC) curve and survival curve of serum *MIR210HG* in CRC patients. (A) The expression of serum *MIR210HG* is of diagnostic value in patients with CRC. (B) The low expression of serum *MIR210HG* has good prognostic value in patients with colorectal cancer.

Receiver operating characteristic (ROC) curve was plotted, and it was determined that the area under curve (AUC) for serum *MIR210HG* in diagnosing CRC was 0.870, accompanied by a sensitivity of 86.5% and a specificity of 71.79% (refer to Figure 2A). By the end of the follow-up, a total of 81 patients were alive and 39 patients had died. The Kaplan–Meier survival assessment revealed that the survival rate of patients with low expression of *MIR210HG* in tumor tissues was 88.46% (46/52), and the survival rate among the tissue samples with high *MIR210HG* expression was 51.47% (35/68). The median survival duration for the 2 patient groups was (53.38 ± 2.51) months and (42.49 ± 2.43) months, respectively, with a statistically significant disparity between the groups (Figure 2B). The Cox regression assessment demonstrated that the *MIR210HG* expression of tumor tissues, lymph node metastasis, tumor node metastasis (TNM) stage, differentiation degree, and invasion degree are influencing variables for the prognosis of colorectal cancer patients, as depicted in Table 3.

### The Influence of *MIR210HG* and miR-1226-3p Suppression on Colorectal Cancer Cell Proliferation, Apoptosis, and Invasion

**Figure 3.**
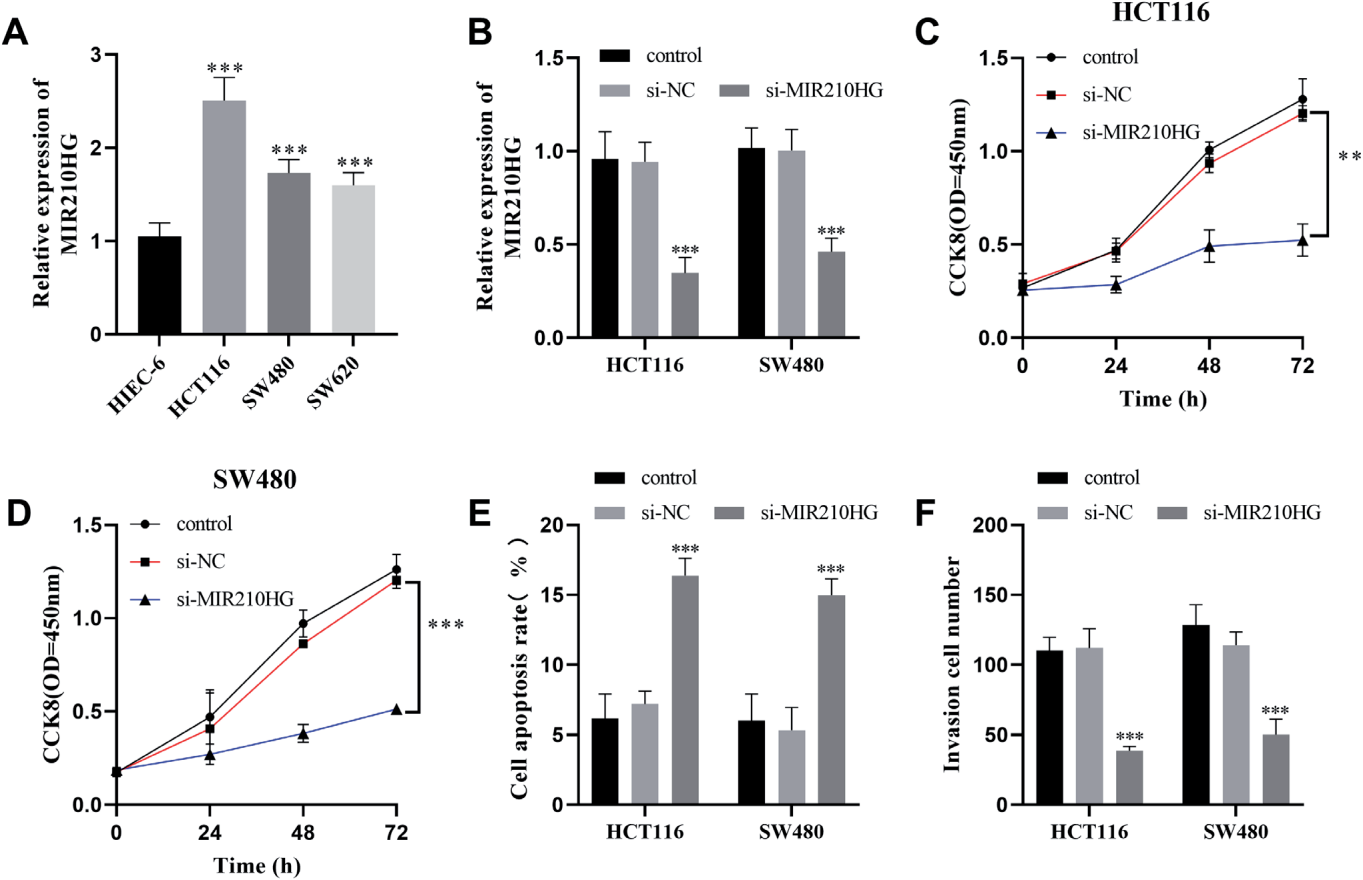
The effect of *MIR210HG* on the function of colorectal cancer cells. (A) The expression of *MIR210HG* elevated in CRC cell lines. (B) The expression level of *MIR210HG* was decreased in CRC cells after knockdown. (C-D) *MIR210HG* knockdown suppressed the cellular growth of CRC cells. (E) Inhibition of *MIR210HG* expression enhanced apoptotic processes in colorectal cancer cells. (F) *MIR210HG* silencing impeded the invasion of CRC cells.^ ***^P < .001, ^**^P < .01.

**Figure 4.**
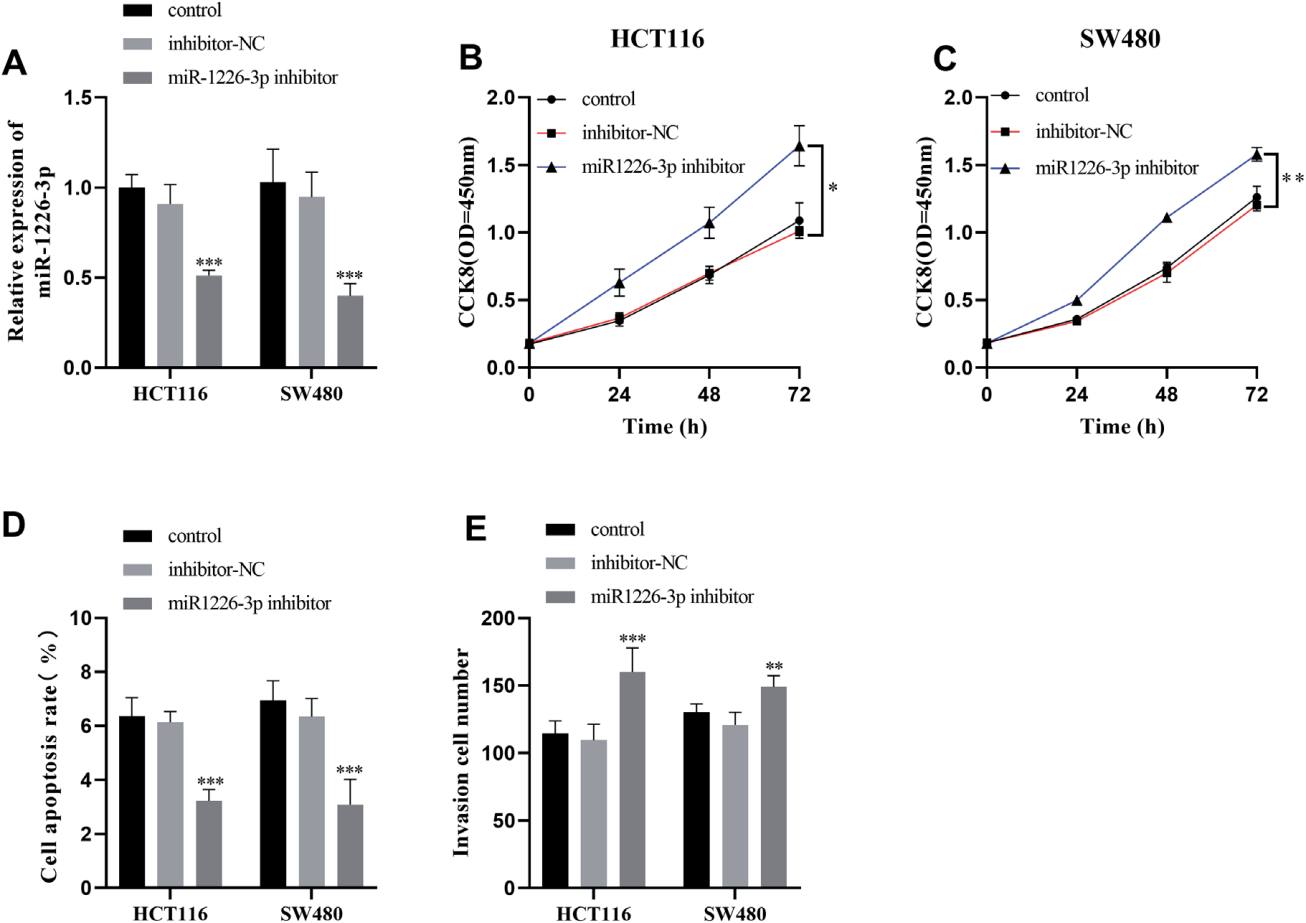
The impact of miR-1226-3p on the function of colorectal cancer cells. (A) The expression level of miR-1226-3p was decreased in CRC cells after silencing. (B-C) Upon miR-1226-3p silencing, CRC cell proliferation was enhanced. (D) Following miR-1226-3p inhibition, the apoptosis ratio of CRC cells was reduced. (E) Following the reduction of miR-1226-3p expression, the invasion capacity of CRC cells was enhanced.^ ***^P < .001, ^**^P < .01, and ^*^P < .05.

*MIR210HG* expression in HCT116, SW480, and SW620 cells was identified. The *MIR210HG* level in HCT116 and SW480 increased more substantially (Figure 3A), so these 2 types of cells were chosen for subsequent experiments. After transfecting si-MIR210HG into CRC cells, a significant reduction in *MIR210HG* expression was observed (Figure 3B), and the cell viability was significantly reduced, as shown in Figure 3C and  D. The apoptosis percentage of HCT116 and SW480 cells was significantly elevated (Figure 3E), and the invasion capacity was noticeably weakened (Figure 3F). Furthermore, subsequent to the silencing of miR-1226-3p in HCT116 and SW480 cells, a notable reduction in miR-1226-3p expression was observed (Figure 4A). This led to an improvement in cell vitality (Figure 4B and  C), a reduction in apoptosis rates (Figure 4D), and an enhancement in invasion capabilities, as depicted in Figure 4E.

### *MIR210HG* and miR-1226-3p have a Targeted Relationship

**Figure 5.**
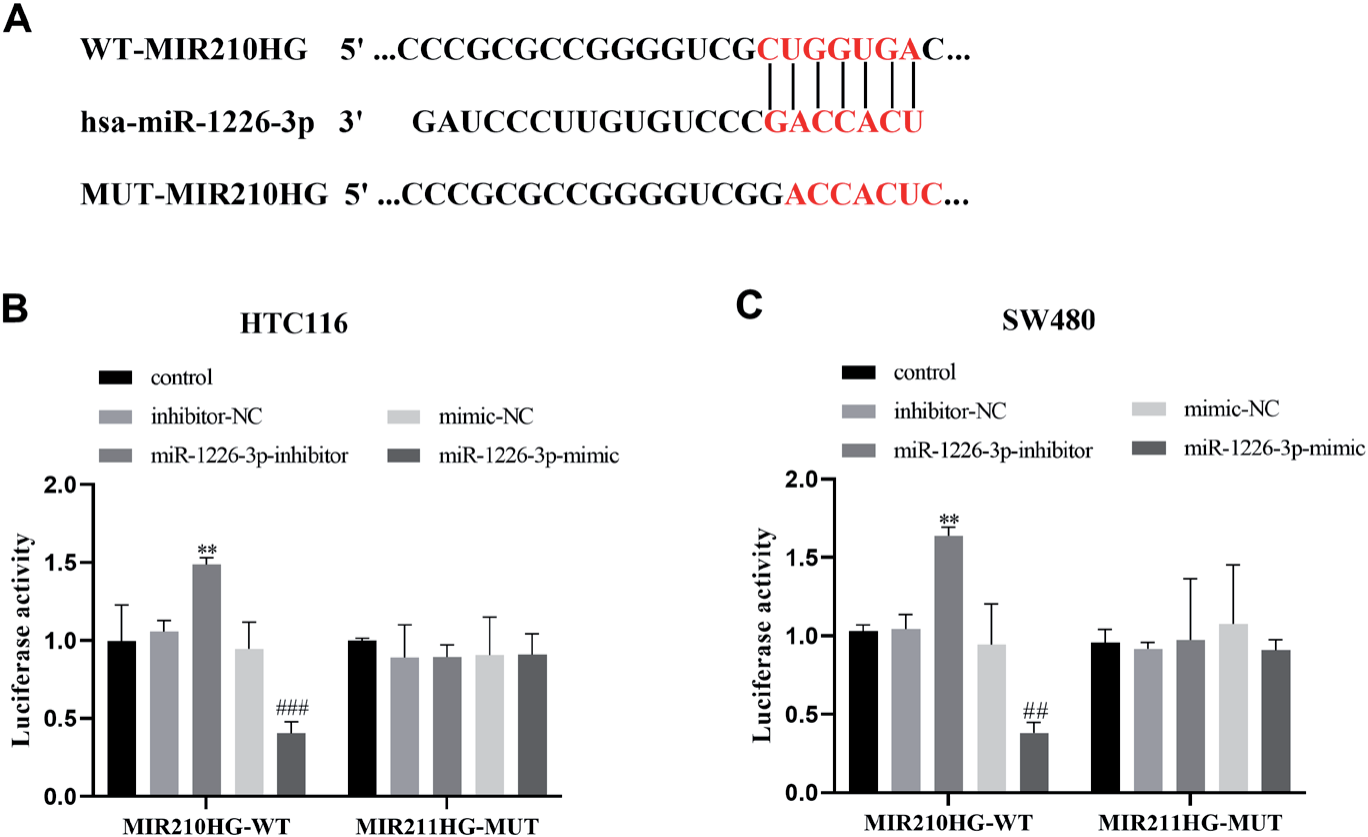
*MIR210HG* can target and regulate the miR-1226-3p. (A) The specific binding regions for *MIR210HG* in the 3’-UTR region of miR-1226-3p. (B-C) miR-1226-3p inhibitor transfection enhanced the luciferase activity of MIR210HG, while miR-1226-3p mimic administration inhibited the luciferase expression of MIR210HG.^ ***^P < .001,^ **^P < .01.

To investigate the involvement of *MIR210HG* in the progression of CRC, the LncRNASNP v3 database (http://gong_lab.hzau.edu.cn/lncRNASNP3/#!/) was employed to forecast the specific binding domains for *MIR210HG* in the 3’-UTR region of miR-1226-3p, as depicted in Figure 5A. After transfecting miR-1226-3p inhibitor in HCT116 and SW480 cells, the fluorescence activity of WT-MIR210HG was significantly elevated. However, in cells exposed to miR-1226-3p mimic, the fluorescence value of WT-MIR210HG was substantially suppressed. The findings are presented in Figure 5B and  C, indicating that *MIR210HG* may play a role in the modulation of miR-1226-3p.

### Inhibition of miR-1226-3p Counteracted the Influence of *MIR210HG* Silencing on Colorectal Cancer

**Figure 6.**
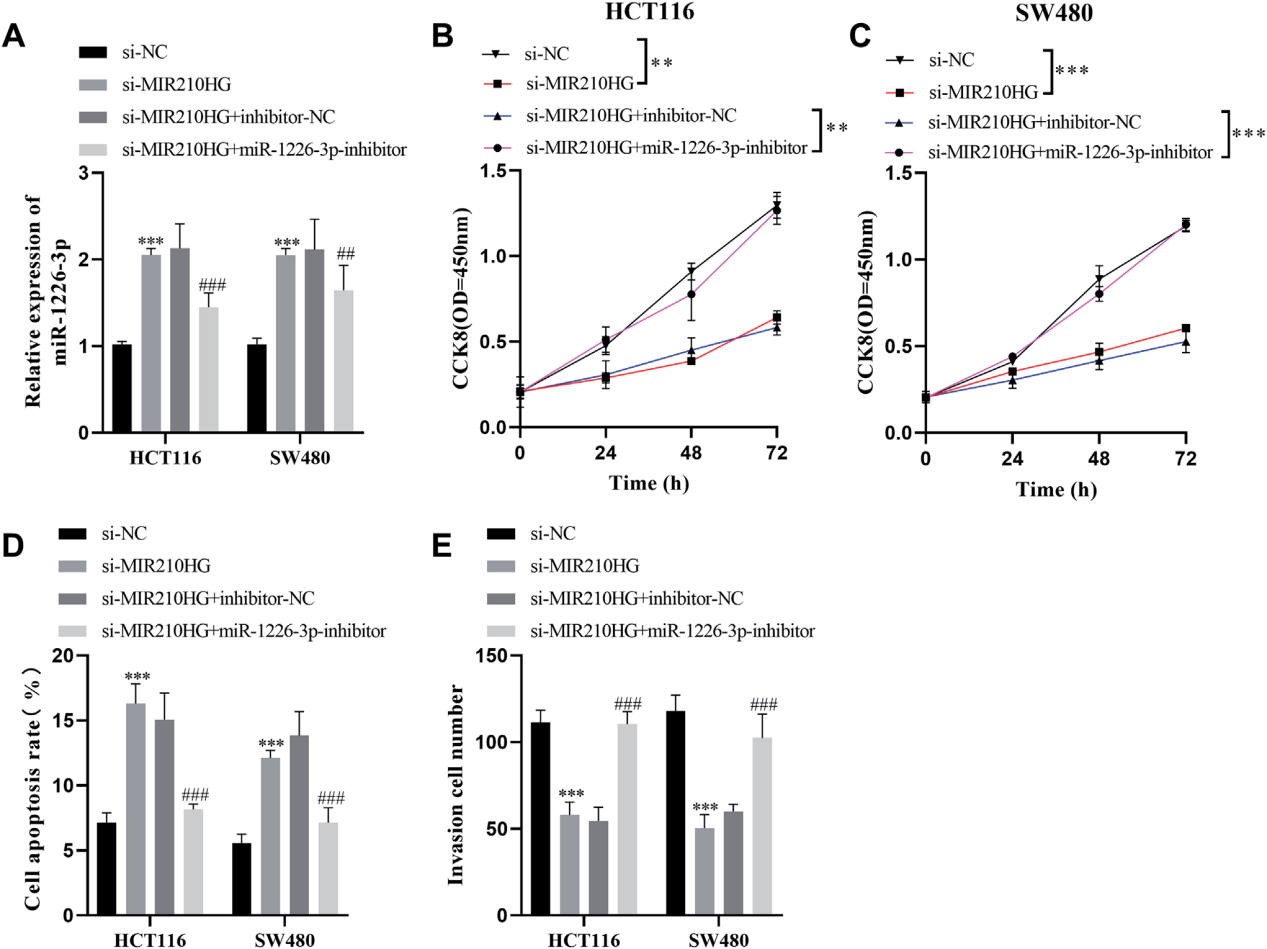
The downregulation of miR-1226-3p reverses the influence of *MIR210HG* inhibition on cell function. (A) Following si-MIR210HG transfection, the miR-1226-3p level was elevated. After transfection with si-MIR210HG and miR-1226-3p inhibitor, the level of miR-1226-3p was reversed. (B-E) Decreased miR-1226-3p expression counters the influence of *MIR210HG* inhibition on cell growth, apoptosis, and invasion. ^**^P < .01, ^*^P < .05.

Subsequently, we verified whether *MIR210HG* controls the growth of CRC by regulating miR-363-3p. Si-MIR210HG significantly increased the miR-1226-3p expression. The miR-1226-3p inhibitor notably diminished the miR-1226-3p when combined with si-MIR210HG (Figure 6A). Cell Counting Kit-8 analysis findings suggested that inhibition of miR-1226-3p counteracted the suppressive influence of *MIR210HG* silencing on cell growth. The findings ara presented in Figure 6B and  C. In the apoptosis experiment, the apoptosis rate was significantly inhibited following transfection with si-MIR210HG and miR-1226-3p inhibitor (Figure 6D). Similarly, the transwell experiment revealed that the miR-1226-3p inhibitor neutralized the inhibitory impact of *MIR210HG* silencing on cell invasion capacity (Figure 6E). Therefore, these data indicated that *MIR210HG* may participate in the modulation of colorectal cancer progression by affecting the expression of miR-1226-3p.

## Discussion

Empirical evidence has elucidated that lncRNAs and miRNAs function either as tumor suppressors or as oncogenes in the etiology and progression of colorectal cancer. For instance, LncRNA GAS5 impedes the progression of CRC by engaging with and enhancing Yes-associated protein (YAP) phosphorylation.^16^ The LINC00460/DHX9/IGF2BP2 interaction complex augments CRC cell proliferation and metastasis by regulating the stability of *HMGA1*.^17^ miR-545 facilitates colorectal cancer progression by hindering the expression of transferrin in the abnormal ferroptosis signaling pathway.^18^ miR-663b facilitates colorectal cancer advancement by stimulating Ras/Raf signaling via the downregulation of TNK1.^19^

lncRNA *MIR210HG* has been shown to contribute significantly to cancer development, for example, cervical cancer, ovarian cancer, pancreatic cancer, and research has shown that *MIR210HG* is linked to the advancement of colorectal cancer.^20,21,22^ The level of *MIR210HG* has prognostic value for CRC patients.^23,24^ To better understand the involvement of *MIR210HG* in CRC, we carried out this research. In our research, we examined the expression of *MIR210HG* in both serum and tumor tissues of CRC patients. The *MIR210HG* expression was upregulated, which also verified the previous conclusions.^23^ Furthermore, we discovered that the *MIR210HG* expression was significantly correlated with serum CEA concentration, lymph node metastasis, TNM stage, tumor size, degree of differentiation, and degree of invasion. Moreover, the expression of *MIR210HG* held important value for the diagnosis of colorectal cancer. Furthermore, we have also confirmed that low *MIR210HG* expression has a favorable prognostic value for patients with colorectal cancer. In previous studies, *MIR210HG* has been demonstrated to enhance the cell vitality and invasion of CRC cells.^20^ Xiaowen Kang et al^25^ have demonstrated that *MIR210HG* is capable of promoting the growth and migration of non-small cell lung cancer cells. Silencing of lncRNA *MIR210HG* suppresses hepatoblastoma cell growth, migration, and invasion through the microRNA-608-FOXO6 pathway.^26^ Here, we proved that *MIR210HG* has the capability to enhance the growth and invasion of CRC cells while suppressing their apoptosis process.

*MIR210HG* promotes tumor metastasis by regulating the expression of mucin-1c in invasive breast cancer, serving as a ceRNA for miR-1226-3p.^15^ Simultaneously, through the lncRNASNP v3 database, we discovered that *MIR210HG* and miR-363-3p possess overlapping binding locations. To demonstrate the targeting relationship between them, we conducted a dual-luciferase reporter gene experiment and found that miR-1226-3p attenuated the luciferase value of MIR210HG. Prior studies have shown that miR-1226-3p is engaged in the development of diverse cancers and executes a crucial function in numerous cancer cells, such as hepatocellular carcinoma, and nasopharyngeal cancer cells.^28^ The hsa-miR-1226-3p was noticeably elevated in gastric tumors yet displayed widespread downregulation in CRC.^27^^,^^29^ We observed that the miR-1226-3p expression was reduced in CRC tissues and serum. Moreover, miR-1226-3p demonstrated the potential to inhibit the proliferation and invasion of colorectal cancer cells and facilitate apoptosis. The interaction between lncRNAs and miRNAs is a frequent occurrence in the field of cancer biology.^30^ In the present study, *MIR210HG* and miR-1226-3p showed a negative correlation. We proved for the first time that knockdown of the 2 in CRC cells also exhibits opposite impacts on cell growth, apoptosis, and invasion. Moreover, in this study, for the first time, we found that suppression of *MIR210HG* expression can block the proliferation and invasion of CRC cells and enhance apoptosis of cells. However, miR-1226-3p silencing reversed these effects. Accordingly, the current investigation has highlighted that *MIR210HG* may contribute to the advancement of colorectal cancer by downregulating the expression of miR-1226-3p.

Through our research, we established that*MIR210HG* may have the capability to accelerate the progression of CRC and alter the functionality of colorectal cancer cells by means of regulating miR-1226-3p. However, we still need to conduct further exploration in the mechanism aspect.

## Conclusion

Our study demonstrated that *MIR210HG* facilitates the development of CRC through the regulation of miR-1226-3p expression and revealed its carcinogenic role in CRC cells. lncRNA MIR210HG/miR-1226-3p may potentially serve as therapeutic targets for addressing colorectal cancer.

## Availability of Data and Materials

The data that support the findings of this study are available on request from the corresponding author.

## Figures and Tables

**Table 1.** Correlation Between the Tumor Tissue Expression of *MIR210HG* and Clinical Indicators in CRC Patients

Parameters		Patients (n = 120)	Low *MIR210HG* Expression (n = 52)	High *MIR210HG* Expression (n = 68)	*P*
Age (years)	< 50	61	28	33	.564
	≥ 50	59	24	35	
Gender	Female	45	18	27	.568
	Male	75	34	41	
Smoking history	No	63	28	35	.796
	Yes	57	24	33	
CEA (ng/mL)	<5	46	25	21	.055
	≥ 5	74	27	47	
Lymph node metastasis	No	91	47	44	.001^**^
	Yes	29	5	24	
TNM stage	I-II	76	40	36	.007^**^
	III-IV	44	12	32	
Tumor size (cm)	<4	54	29	25	.038^*^
	≥ 4	66	23	43	
Tumor differentiation	Poor and moderate	69	24	45	.028^*^
	Well	51	28	23	
Tumor invasion	Low	68	38	30	.002^**^
	High	52	14	38	

**Table 2.** Correlation Between Serum Expression of *MIR210HG* and Clinical Indicators in CRC Patients

Parameters		Patients (n = 120)	Low *MIR210HG* Expression (n = 54)	High *MIR210HG* Expression (n = 66)	*P*
Age (years)	< 50	61	29	32	.569
	≥ 50	59	25	34	
Gender	Female	45	18	27	.394
	Male	75	36	39	
Smoking history	No	63	29	34	.811
	Yes	57	25	32	
CEA (ng/mL)	<5	46	26	20	.045^*^
	≥ 5	74	28	46	
Lymph node metastasis	No	91	48	43	.003^**^
	Yes	29	6	23	
TNM stage	I-II	76	42	34	.003^**^
	III-IV	44	12	32	
Tumor size (cm)	＜4	54	30	24	.036^*^
	≥ 4	66	24	42	
Tumor differentiation	Poor and moderate	69	25	44	.025^*^
	Well	51	29	22	
Tumor invasion	Low	68	39	29	.002^**^
	High	52	15	37	

CEA, serum carcinoembryonic antigen; TNM, tumor node metastasis. ^*^P＜0.05, ^**^P＜0.01.

**Table 3.** Cox Regression Analysis of Prognostic Factors in CRC Patients

Parameters	*P*	HR	95% CI
Tumor tissue *MIR210HG* expression	.014^*^	3.927	1.318-11.699
Age	.222	0.648	0.323-1.300
Gender	.455	0.762	0.373-1.556
Smoking history	.522	1.300	0.582-2.904
CEA	.504	0.764	0.348-1.681
Lymph node metastasis	.001^**^	0.298	0.143-0.618
TNM stage	.016^*^	0.395	0.186-0.839
Tumor size	.074	0.409	0.153-1.092
Tumor differentiation	.008^**^	0.349	0.160-0.764
Tumor invasion	.042^*^	0.400	0.165-0.969

CI, confidence interval; HR, hazard rate; CEA, serum carcinoembryonic antigen; TNM, tumor node metastasis. ^*^P＜0.05, ^**^P＜0.01.
